# Do health and social support and personal autonomy have an influence on the health-related quality of life of individuals with intellectual disability?

**DOI:** 10.1186/s12913-018-3856-5

**Published:** 2019-01-23

**Authors:** Montserrat Alonso-Sardón, Helena Iglesias-de-Sena, Luz Celia Fernández-Martín, José Antonio Mirón-Canelo

**Affiliations:** 1 Area of Preventive Medicine and Public Health, School of Medicine, University of Salamanca, Institute of Biomedical Research of Salamanca, Avd de Alfonso X El Sabio s/n, Facultad de Medicina, Universidad de Salamanca, 37007 Salamanca, Spain; 2 Day Psychiatric Hospital of the University Clinic Hospital of Salamanca, Salamanca, Spain

**Keywords:** Personal autonomy, Perceived social support, Health-related quality of life, Individuals with intellectual disability

## Abstract

**Background:**

The aim of this study was to assess the relation between perceived social support and personal autonomy of individuals with intellectual disabilities and Health-Related Quality of Life.

**Methods:**

A cross-sectional study with a multicentre sample was carried out including 162 institutionalized individuals with intellectual disability. The measurement tool was a structured questionnaire with *sociodemographic variables*, and three scales: *Functional Independence Measure*(FIM) *scale*, *Duke-UNC Functional Social Support Questionnaire*, and *SF-36 Health Survey*, which were completed during an individual/family interview.

**Results:**

The perception of *received social support* is high on all 11 items of the Duke-UNC questionnaire, with an average of 3.45 for item-1 and 4.85 for item-11, which represents a *total perceived support* of an average 47.98 points (±SD7.30) (normal support). The Mental-Health component is rated worse than Physical-Health (67.41 vs. 71.74). The average rates for the different dimensions range from 57.34 points for Social-Functioning (the lowest rating) to 79.61 points for Bodily-Pain (highest rating). A multiple linear regression analysis reveals that the dimensions of Physical-Functioning (*p* < 0.001), Role-Physical (*p* = 0.016) and Bodily-Pain (*p* = 0.022), which are elements of the Physical-Health component, are independent predictive variables with the Degree of Autonomy (FIM) as a dependent variable. Social-Support (*Duke-UNC*) as a dependent variable is determined by the dimensions of Vitality (*p* = 0.014), Role-Emotional (*p* = 0.001) and Mental-Health (*p* < 0.001), which are part of the Mental-Health component and act as independent predictive variables.

**Conclusions:**

Individuals with intellectual disability and a higher degree of *personal autonomy* determined by institutional and family support report better Health and Quality of Life.

**Electronic supplementary material:**

The online version of this article (10.1186/s12913-018-3856-5) contains supplementary material, which is available to authorized users.

## Background

People with disabilities are an important population group which in 2008 represented 8.5% (3,847,900 people) of the Spanish population. This quantitative impact makes it necessary to assess the major healthcare and social challenges, as well as the social and healthcare costs which it entails, given the levels of dependence of these people for families and for Society [1, 2].

Social support has been significantly associated to the Health-Disease process, and the perception of social support by individuals and their relatives has been systematically related to a good health. On the contrary, dysfunctional or discontinuous social support, or its absence, increases the vulnerability of patients to health problems and diseases. Also, the influence of social support has been observed on other positive health indicators, such as Well-being [3], perceived Health [4] and Quality of Life [5]. Perceived health improves health-related quality of life (HRQoL), and adding social support can improve Well-being, mainly physical and emotional.

Most healthcare professionals are aware that it is necessary to implement a comprehensive policy if we want socio-health care to meet the needs and demands of the most vulnerable social groups in order to achieve a higher degree of integration which guarantees equality of opportunities and equity for these population groups [6]. In this regard, and if we want this inclusion to be effective, it is necessary to obtain the highest possible individual autonomy through the achievement of the maximum development of their physical, cognitive and emotional abilities and/or competences, thus creating adequate and normalized social dynamics which enable individuals with intellectual disability (IIDs) to carry out their basic daily activities and prevent deficiencies or limitations in their activity which may restrict their participation and have an influence on or lead to additional problems and/or dependence.

In order to work in these three professional areas, the approach needs to be multidisciplinary, as an essential tool in the education of IIDs or individuals with developmental disability. For this reason, caregivers, educators, physicians, physical therapists, occupational therapists, speech therapists, psychologists and other professionals must work as a team to correct or minimize those alterations as best as possible, and to guarantee the maximum individual autonomy and social integration so that any person with any kind of disability may have the possibility to come as close as possible to the standard levels of well-being and health-related quality of life (HRQoL) [7] of the general population.

The **objective** of this social and applied research has been to assess the association and influence of comprehensive care and social support services received by institutionalized IIDs on the improvement of their individual autonomy, their well-being and their HRQoL. With this, the authors intend to increase and improve the evidence on the importance that supports and social assistance have on the quality of life and well-being of this population group.

## Methods

### Study design

In order to reach the objectives, we performed a multicentric *cross-sectional population-based descriptive study* which was carried out through a *personal interview to people with intellectual disability with the support of their caregivers* and in the company of their relatives and/or legal guardians; but with prevalence of the opinion of IIDs. In the interviews, caregivers and social workers collaborated together with the purpose of improving the understanding of the questions and thus improve the reliability and validity of the answers.

### Sample/participants

The population of the study are institutionalized IIDs who live in residential homes and attend occupational and/or leisure centres in three Spanish provinces (Salamanca, Zamora and Cáceres), who receive comprehensive health and occupational care in their residence and/or home or only during daytime, from 9 a.m. to 5 p.m. Criteria for inclusion in the study included obtaining *informed consent* by the institutionalized subjects who would participate in the study, either given by the IIDs themselves or by their relatives, as well as receiving an authorization by the director of the centre in which they are institutionalized (see Additional file 1). The sample was randomly selected and it returned 162 people with a very similar distribution in the three centres: 55, 51 and 46 people, respectively.

### Data collection procedures/tools

The measuring instrument used was a *structured questionnaire*, which was completed during an *individual/family interview* and which includes, on the one hand, *sociodemographic variables* which will make it possible to characterize the sample (age, sex, percentage of disability, cause of disability, environment and place of residence); and on the other hand the scales for the assessment of function and quality of life parameters which are being studied: *Functional Independence Measure* (FIM) *scale* [8], *Duke-UNC Functional Social Support Questionnaire* [9, 10], and the *SF-36 Health Survey* [11–14].

The *Functional Independence Measure* (FIM) *scale* is a globally accepted tool to measure the degree of disability and the independence of the patients for their activities of daily living (ADL). The scale measures 18 activities which are classified into two dimensions, *physical* and *cognitive*, and which are in turn divided into 6 categories which measure self-care, sphincter control, transfer, locomotion, communication and social cognition. Each item is scored from 1 to 7, ranging from total dependence to independence, and the total score ranges from 18 points (total dependence) to 126 points (complete independence), so that the lower the score, the worse the functional level of the patient [1, 2]. In this study, we have chosen the FIM scale, because the variables it measures include cognitive aspects, communication and interaction with the environment, which are key elements in the life of IIDs. The FIM scale is currently a standard in the global literature which is widely used for different pathologies and age groups, and which has proven to be a valid, sensitive and reliable instrument for the functional assessment of disability.

The *Duke-UNC Functional Social Support Scale* is a simple and brief tool which assesses the perceived functional or qualitative social support, adapted to the Spanish population and widely used in the caregiver community [8, 9]. This scale includes 11 items which are scored on a Likert-like scale ranging from 1-“*Much less than I would like*” to 5-“*As much as I would like*”; so that the final score is a quantitative measure of the perceived social support: the lower the score, the less the perceived support. The score range of global functional support moves between 11 and 55 points, and the cut-off score to consider that there is perceived support is < 32 points. The scale is divided into two subscales: *confidential* social support, which represents the possibility to communicate with other people (items 1, 4, 6, 7, 8, 10); and *affective* social support, which assesses the received displays of affection and empathy, as well as the availability of people for those displays of affection (items 2, 3, 5, 9, 11) [9, 10]. It has a grade B of recommendation according to experts [15].

Quality of life was measured with the *SF-36 Health Survey*, the most widely used questionnaire globally which assesses health-related quality of life in terms of physical and psychological functioning through eight dimensions: Physical Functioning-PF (physical limitations), Role Physical-RP (interference with work and daily activities), Bodily Pain-BP (intensity of pain and its effect on activities), General Health-GH (personal assessment of health), Vitality-VT (feeling of energy), Social Functioning-SF (interference with normal social activities), Role Emotional-RE (interference with work or other daily activities) and Mental Health-MH (depression, anxiety, control of emotions and behaviour). It is expressed as a score on a 0–100 scale in which higher scores represent better quality of life. This questionnaire has a grade A of recommendation according to experts [15] and has been used with people with low levels of education and IIDs [16–18]. HRQoL with social supports improves individual functioning and, consequently, improves both physical and emotional Well-being.

The fieldwork was carried out during the last quarter of the year 2015 and the first quarter of the year 2016. The interviews were made in the centers where IIDs are institutionalized and always in the presence of the caregivers and social workers.

The *personal interview* as a social research technique makes it possible to obtain the desired information from a specific subject beforehand through a direct conversation, based on a *structured questionnaire*. That is, all the subjects interviewed are asked the same questions, in the same way, and in the same order. Therefore, the questionnaire was applied to the selected IIDs in front of their families or of professionals from supporting institutions in cases in which the subject had a greater dependence. In all cases, questions were adapted to the level of understanding of IIDs by social workers. They have great experience in dealing with IIDs. In this way, we try to control bias and improve the validity of the study.

### Ethics

This study was reviewed and approved by the *Clinical Research Ethics Committee for Clinical Investigation of the University Hospital of Salamanca* (see Additional file 2).

### Statistical analyses

The data obtained were included in a database which had been specifically created for this research, and they were analysed with the statistical software package SPSS 23.0. The statistical analysis included a descriptive study of frequency distribution of all variables (*univariate analysis*). Quantitative results are expressed as *arithmetic mean* and *standard deviation* (SD), qualitative results are expressed as relative frequencies (percentage or *proportion*), accompanied by their corresponding *95% confidence intervals* (95% CI), which make it possible to assess the population parameter through the values observed in the different variables of the individuals in the sample. On the other hand, a *multiple linear regression model* was used to explore and quantify the relation between the scores (quantitative variables) obtained in the different scales/questionnaires of functional assessment and quality of life that were applied (*multivariate analysis*). The results are included in simplified tables which show the regression coefficient (B), 95% CI for B and statistical significance (p). Finally, the researchers in this study have defined a *p*-value for statistical significance of *p < 0.05*.

## Results

The study includes 162 adult patients, 58% (94) are men and 42% (68) are women. The average age is 50.8 years ± SD 12.85 (range: 21-80 years). Most of the subjects live in residential homes and go to occupational and/or leisure centres (112; 69.1%). The degree of disability of these people ranges from 36 to 100%, and the average is 76.17% ± SD 10.38.

Tables 1 and 2 show the qualitative and quantitative results, respectively, which were obtained in the *FIM Scale*. As can be seen in Table 1, with regard to *Self-care*, the subjects did not generally require care (levels 6 and 7) for each of the functions analysed, with percentages ranging from 53.1% (eating) to 32.7% (bathing/showering). Also, bathing/showering is the activity which more often requires total assistance (levels 1 and 2), with 29%. Most of the subjects can *control their sphincters*. 66–67% of the subjects do not require help in this regard (levels 6 and 7), while only 10% show complete dependence (levels 1 and 2). With regard to *mobility and locomotion*, most of them do not require help (levels 6 and 7), with percentages ranging from 59.3% (transfers: bathtub/shower) to 70.4% (transfers: bed/chair). *Communication* and *cognition* are the functions with the highest levels of complete (levels 1 and 2) and partial dependence (levels 3, 4 and 5), and problem solving scores in some of these levels in the highest proportion (81.5%). Therefore, only 18.5% of the patients consider themselves to be able to solve problems.

**Table 1:** Main qualitative results obtained in the FIM scale

FIM	Categories	Complete dependence		Modified dependence		No assistance	
		Levels 1,2		Levels 3,4,5		Level 6,7	
		F	% ± 95%CI	F	% ± 95%CI	F	% ± 95%CI
Self-care	Eating	20	12.3 ± 5	56	34.6 ± 7	86	53.1 ± 8
	Grooming	25	15.4 ± 6	55	34 ± 7	82	50.6 ± 8
	Bathing/showering	47	29 ± 7	62	38.3 ± 7	53	32.7 ± 7
	Dressing upper body	28	17.3 ± 6	56	34.6 ± 7	78	48.1 ± 8
	Dressing lower body	29	17.9 ± 6	53	32.7 ± 7	80	49.4 ± 8
	Toileting	45	27.8 ± 7	63	38.9 ± 8	54	33.3 ± 7
Sphincter control	Bladder	16	9.9 ± 5	37	22.8 ± 6	109	67.3 ± 7
	Bowel	14	8.6 ± 4	40	24.7 ± 7	108	66.7 ± 7
Mobility	Bed/chair	19	11.7 ± 5	29	17.9 ± 6	114	70.4 ± 7
	Toilet	18	11.1 ± 5	31	19.1 ± 6	113	69.8 ± 7
	Bathtub/shower	25	15.4 ± 6	41	25.3 ± 7	96	59.3 ± 8
Transfers	Walking/wheelchair	19	11.7 ± 5	29	17.9 ± 6	114	70.4 ± 7
	Stairs	23	14.2 ± 5	34	21 ± 6	105	64.8 ± 7
Communication	Comprehension	24	14.8 ± 5	84	51.9 ± 8	54	33.3 ± 7
	Expression	35	21.6 ± 6	79	48.8 ± 8	48	29.6 ± 7
Social cognition	Social interaction	38	23.5 ± 7	80	49.4 ± 8	44	27.2 ± 7
	Problem solving	65	40.1 ± 8	67	41.4 ± 8	30	18.5 ± 6
	Memory	50	30.9 ± 7	70	43.2 ± 8	42	25.9 ± 7

**Table 2:** Main quantitative results obtained in the FIM scale

FIM		Categories		Subscales		Domains		Total FIM	
		Mean	± SD	Mean	± SD	Mean	± SD	Mean	± SD
Self-care	Eating	5.25	± 1.85	Self-care *(max. 35 points)*		Motor *(max. 91 points)*		Total *(max. 126 points)*	
	Grooming	5.03	± 2.02						
	Bathing/showering	4.15	± 2.23						
	Dressing upper body	4.94	± 2.06	28.30	± 11.51				
	Dressing lower body	4.94	± 2.11						
	Toileting	4.17	± 2.25						
Sphincter control	Bladder	5.63	± 1.85	Sphincter c. *(max. 14 points)*					
	Bowel	5.71	± 1.76	11.31	± 3.60	66.59	± 23.28		
Mobility	Bed/chair	5.67	± 1.92	Mobility *(max. 21 points)*					
	Toilet	5.68	± 1.90	16.65	± 5.68			86.65	± 30.28
	Bathtub/shower	5.27	± 2.06						
Transfers	Walking/wheelchair	5.69	± 1.87	Transfers *(max. 14 points)*					
	Stairs	5.43	± 2.05	11.17	± 3.93				
Communication	Comprehension	4.33	± 1.77	Communication *(max. 14 points)*		Cognitive *(max. 35 points)*			
	Expression	4.17	± 1.89	8.62	± 3.64				
Social cognition	Social interaction	4.06	± 1.86	Social cog. *(max. 21 points)*					
	Problem solving	3.31	± 1.89	11.09	± 5.34	20.17	± 10.25		
	Memory	3.75	± 1.94						

Table 2 shows the quantitative data obtained in the different categories, sub-scales and dimensions. It may be highlighted that the average score for physical dimension was 66.59 (± SD 23.28) out of a total of 91 points, whereas the score for the cognitive dimension is 20.17 (± SD 10.25), out of a maximum of 35 points.

The results obtained in the *Duke-UNC Functional Social Support Scale* are presented in Table 3. In general terms, we may highlight that there is a high perception of received social support in the 11 items of the questionnaire (4 “*Almost as much as I would like*” and 5 “*As much as I would like*” in the Likert scale), with average scores ranging from 3.45 in item 1 “*I receive visits from friends and relatives*” to 4.85 in item 11 “*I get help when I am sick in bed*”, which represents an average total perceived support of 47.98 points (± SD 7.30) (normal support), which is much higher than the established cut-off value (32 points). In 154 out of the 162 IIDs in our study (95%), the perceived social support was normal. A specific analysis of the confidential and affective support reveals an average of 25.71 (±SD 4.69) and 22.40 (± SD 2.97) points, respectively, and these scores are higher than the established cut-off values (18 points for confidential support and 15 points for affective support).

**Table 3:** Main results obtained in the DUKE-UNC Functional Social Support

DUKE-UNC Functional Social Support		Descriptive study of frequencies					Descriptive statistics
		1	2	3	4	5	
		Much less than I would like	Less than I would like	Some, but would like more	Almost as much as I would like	As much as I would like	
		Freq. (%)	Freq. (%)	Freq. (%)	Freq. (%)	Freq. (%)	Mean(±SD)
1	I receive visits from my friends and relatives	30 (18.5%)	22 (13.6%)	19 (11.7%)	27 (16.7%)	64 (39.5%)	3.45 (±1.56)
2	I receive help around the house	4 (2.5%)	9 (5.6%)	23 (14.2%)	42 (25.9%)	84 (51.9%)	4.19 (±1.03)
3	I receive praise for a good job		4 (2.5%)	6 (3.7%)	45 (27.8%)	107 (66.0%)	4.57 (±0.68)
4	I have people who care what happens to me		3 (1.9%)	8 (4.9%)	30 (18.5%)	121 (74.7%)	4.66 (±0.66)
5	I get love and affection		9 (5.6%)	4 (2.5%)	40 (24.7%)	109 (67.3%)	4.53 (±0.79)
6	I get chances to talk to someone about problems at work or with my housework	3 (1.9%)	9 (5.6%)	8 (4.9%)	40 (24.7%)	102 (63.0%)	4.41 (±0.94)
7	I get chances to talk to someone about my personal or family problems	1 (0.6%)	9 (5.6%)	15 (9.3%)	36 (22.2%)	101 (62.3%)	4.40 (±0.91)
8	I get chances to talk to someone about money matters		11 (6.8%)	18 (11.1%)	36 (22.2%)	97 (59.9%)	4.35 (±0.92)
9	I get invitations to go out and do things with other people		13 (8.0%)	17 (10.5%)	49 (30.2%)	83 (51.2%)	4.24 (±0.93)
10	I get useful advice about important things in life		5 (3.1%)	17 (10.5%)	42 (25.9%)	98 (60.5%)	4.43 (±0.80)
11	I get help when I am sick in bed		2 (1.2%)	1 (0.6%)	15 (9.3%)	144 (88.9%)	4.85 (±0.45)
Total Perceived Support		*≤ 32 points-Low support; ≥ 33 points-Normal support*					47.98 (±7.30)

Table 4 includes the descriptive statistics for each of the health components and dimensions of the *SF-36 Survey*. In general terms, the Mental Health component received worse scores than the Physical Health component (67.41 vs. 71.74). The average scores of the dimensions range from 57.34 points for Social Functioning (lowest rating) to 79.61 points for Bodily Pain (highest rating).

**Table 4:** Main results obtained in the SF-36

SF-36 Dimensions	Descriptive statistics	
	Mean	± Standard Dev.
Physical Functioning (PF)	71.55	33.79
Role-Physical (RP)	75.30	41.13
Bodily Pain (BP)	79.61	27.08
General Health (GH)	59.32	21.03
Physical health COMPONENT (PHC)	71.74	25.68
Vitality (VT)	69.25	20.30
Social Functioning (SF)	57.34	21.61
Role-Emotional (RE)	69.13	40.96
Mental Health (MH)	73.92	19.85
Mental health COMPONENT (MHC)	67.41	18.91
Health Transition Item	48.46	17.55

**Fig. 1:**
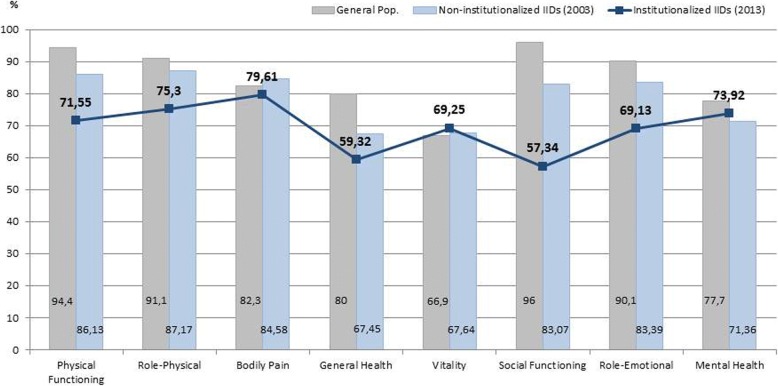
Values obtained in the institutionalized population with intellectual disability with the reference values of the general population and the values of their non-institutionalized peers

Figure 1 compares the values obtained in the institutionalized population with intellectual disability with the reference values of the general population and the values of their non-institutionalized peers. The largest differences are observed in the Social Functioning component, both between the two groups of IIDs (institutionalized and non-institutionalized) and with regard to the general population of reference.

The association study between the sociodemographic variables included and the different scales/surveys of functional assessment and HRQoL used in the study reveals some results which are worth highlighting. The independent variable *sex* is not significantly associated (*p* > 0.05) to any of the scales, sub-scales, domains or components of the different surveys and/or scales, whereas the variable *age* is significantly associated to the perceived social support in the *Duke-UNC questionnaire* (*p* = 0.027) and to some dimensions of the *SF-36 Survey*: Physical Functioning (*p* = 0.029), Vitality (*p* = 0.009) and Mental Health (*p* = 0.045). With regard to the independent variable *rural/urban origin*, it is significantly associated to perceived social support in the *Duke-UNC Questionnaire* (*p* < 0.001), the cognitive domain of the *FIM Scale* (*p* = 0.015) and some dimensions of the *SF-36 Survey*: Physical Functioning (*p* = 0.012), General Health (*p* = 0.016), Vitality (*p* = 0.001) and Mental Health (p = 0.001). With regard to the *degree of disability*, it was significantly associated to all the sub-scales and domains of the *FIM Scale* (*p* < 0.05), but not with the perceived social support of the *Duke-UNC Questionnaire* or any of the dimensions of the *SF-36 Survey* (*p* > 0.05).

The multiple linear regression analysis with the *FIM Scale* as a dependent variable reveals that the dimensions of Physical Functioning (*p* < 0.001), Role Physical (p = 0.016) and Bodily Pain (*p* = 0.022), which are elements of the Physical Health component, are independent predictive variables. On the other hand, an analysis with the *Duke-UNC Questionnaire* as a dependent variable shows that the dimensions of Vitality (*p* = 0.014), Role-Emotional (*p* = 0.001) and Mental Health (p < 0.001), which belong to the Mental Health component, act as independent predictive variables (see Table 5).

**Table 5:** Multiple linear regression analysis between FIM scale and Duke-UNC *(dependent variables)* and the Dimensions of SF-36 *(predictive variables)*

Predictive variables		Dependent variable							
Dimensions SF-36		Total FIM				Duke-UNC Total perceived support			
		B	95% CI for B		Sig.	B	95% CI for B		Sig.
			Lower l.	Upper l.			Lower l.	Upper l.	
Physical Health Component (PHC)	Physical Functioning	0.543	0.386	0.701	0.000*	0.013	−0.028	0.054	0.528
	Role-Physical	0.180	0.033	0.327	0.016*	−0.011	−0.049	0.027	0.562
	Bodily Pain	−0.287	−0.532	−0.042	0.022*	− 0.096	−0.159	− 0.033	0.003*
	General Health	0.063	−0.227	0.353	0.666	0.032	−0.043	0.107	0.406
Mental Health Component (MHC)	Vitality	−0.261	−0.630	0.108	0.164	0.120	0.025	0.216	0.014*
	Social Functioning	0.087	−0.096	0.271	0.348	−0.018	−0.065	0.029	0.455
	Role-Emotional	0.118	−0.004	0.239	0.059	−0.054	−0.085	− 0.022	0.001*
	Mental Health	0.040	−0.289	0.369	0.810	0.162	0.077	0.247	0.000*

*Statistical significance level of 5% (*p* < 0.05)

## Discussion

Our findings show that the IIDs in our study report an adequate level of *Perceived Social Support (PSS)*, and that families are the main providers of that support. These results are explained by the role traditionally played by families in Spain. These observations are in keeping with those of other authors which claim that family support is associated to a better perception of quality of life, although we are aware that this role may be overvalued compared with other types of social support [19].

Most of the participants in the study have a good family support, which represents a mechanism of protection and tutelage against stressful situations, since the data reveal that a normal functioning family network fulfils a social function and a social responsibility. Recent studies carried out in Spain highlight the relevant role of families, which have beneficial effects during adult and old age because they make it possible to manage stressful situations and they act as a protective element [20]. This role is beginning to be assessed with new tools, and it is therefore still necessary to establish the validity and reliability of these results [21].

In this study, the sample of the analysis is made up of IIDs who live in institutional residential centres and attend occupational and/or leisure centres with similar human and professional resources for their care, since they all belong to the same federation of associations of IIDs (FEAPS). Therefore, they all have the same aim and organic and functional structure. Consequently, users receive virtually constant support and supervision by their caregivers and educators, in spite of the fact that in some cases it may not be really necessary. We are aware that this factor may have an influence on the results, since it reduces the real level of independence of each subject; however, it is necessary to assess the adaptation and effectiveness of these centres with regard to their main aim, which is to improve the Quality of Life of IIDs. This is an aspect which is beginning to be assessed in other countries as well [22]. With regard to non-institutionalized IIDs, significant differences are observed with regard to the factors of Physical Functioning, General Health, Social Functioning and Role-Emotional, probably due to a worse situation and to the fact that they spend more time in the centres and less time is dedicated to other interpersonal and social relationships [17]. This result is coherent, since the components of Physical Functioning and Role-Physical are determinant factors for General Health and Vitality. On the other hand, Social Functioning is conditioned by these elements, but mainly by the attitude of the parents and guardians. With regard to the Mental Health component, which shows a similar level in both populations, this finding may be due to a lack of understanding and objective judgement on the implications of this component [17].

With regard to the relation between *social functioning* and *emotional state*, the sample is characterized by the possibility of communicating with and showing affection or empathy to other people, which makes it possible to attain a dynamic balance in their emotional state. Consequently, social support is related to their emotional well-being, and it has been proven that the more perceived social support there is, the less emotional problems will the older IID develop. The results confirm the important role played by social support and services on *emotional state and perceived health* during adult age. The subjects who live within a community context enjoy a better psychosocial adjustment, which is reflected in a higher participatory and perceived social support, whereas residents without social support report a poorer state of physical and emotional health, with low levels of self-esteem, and with friends as the main source of support. This aspect has been highlighted by a recently published systematic review in relation with the general population [23].

This finding confirms the existing relation between life conditions and personal satisfaction which is influenced by personal values and determines the quality of life of people. Therefore, it may be claimed that better life conditions lead to a better quality of life; and that the higher the personal satisfaction, the higher the Quality of Life, and that the more culture and maturity (*personal values*), the higher the Quality of Life [11, 14].

All these findings have been proven in a sample of institutionalized and non-institutionalized subjects over 65 years old, in which it was observed that the relational aspect was the most relevant one in non-institutionalized elderly people, and that they derived a high perception of satisfaction through family support, whereas for institutionalized subjects, the main source of support were social relational aspects [24]. The positive effect provided by PSS is clear in the general population: these benefits represent higher well-being for elderly people and their families, decrease the feelings of isolation and promote healthy behaviour. More specifically, in the field of Health, social support is important to face stress and disease, but we must be aware that in IIDs, supportive interpersonal and social relations may be both positive and negative, as some studies have reported [25]. Social support has been presented as a useful element in the maintenance of Health and to prevent the adjustment disorders and psychopathologies which are characteristic of IIDs [26]. It may also play an important role in the reduced prevalence of some classic risk factors, such as tobacco and alcohol. This last factor has not been widely researched in IIDs because families tend to be overprotective and reduce the consumption of these substances [27]; but some essays are starting to assess support interventions to reduce their consumption [28]. On the other hand, some recent studies claim that social support does not replace formal healthcare with regard to Health, but that it is a complement for it [29].

Finally, with regard to the PSS measured by the Duke-UNC questionnaire, we have observed a favourable assessment of their social relations (relatives and close friends), probably related to the possibility of maintaining an empathic and emotive communication with them, both at an affective and at a confidential level. It may be claimed that the *level of affective and confidential support* and the *total perceived support level* of the sample are relevant because they largely exceed the established value of reference from a healthcare perspective. In our country, there are only studies of interventions with caregivers of dependent people, elderly people and immobilized patients at their homes [30–32].

It may be added that based on what we have observed through the SF-36 Survey in this sample of IIDs, this deficit in cognitive knowledge protects the patients against the current chronic and/or neurodegenerative diseases which are associated to the process of ageing and which represent the biggest concern in the general population. The cognitive deficit of IIDs with regard to the concepts of Health-Disease acts as a simple protection in which they are not aware of the transcendence of certain ailments [33, 34]. This mechanism probably works similarly to the way in which their relatives protect them from classic risk factors like tobacco and alcohol consumption and to the way in which their family and social support assist them in their daily life to meet the social integration challenges and needs that may arise [16, 27].

### Limitations and strengths

The most important **limitation** in this study is related to the difficulty of IIDs to understand the items of the sub-scales or the components of the SF-36 Survey of Health-Related Quality of Life. As an example, the *Mental Health* component, which is already difficult to understand for a normal person, is much more elusive for IIDs who lack the objective judgement to understand its meaning and to assess their own mental health. This limitation may account for the fact that their levels are similar to those of the general population of reference. We have attempted to control this limitation through the support of their caregivers, who know them perfectly and who report that the subjects are IIDs but know what is happening to them and what is good for them, that they are not stupid and are capable of understanding and assimilating information, and of acting accordingly [35].

The authors are aware of these limitations and we consider that the main **strength** of this multicentric study is to simultaneously use three measurement instruments that are usually used independently in most of the studies reviewed. In this way, we want to show a more objective view of HRQoL in IIDs.

## Conclusions

In conclusion, we may declare that Individuals with Intellectual Disability with a higher degree of Personal Autonomy associated to received and perceived family and institutional support report a significantly higher General Health, Well-being and HRQoL than subjects who are more dependent and have less support.

Finally, it will be necessary to carry out future intervention studies that combine qualitative and quantitative methodologies to assess HRQoL, PSS and Autonomy in people with different degrees and types of disability in order to enact the Rights established in the UN Convention on the Rights of Persons with Disabilities and, consequently, to provide a better Social Function and Integration.

## Additional files

Additional file 1: Statement of parental/legal guardian consent to participate (PDF 170 kb)

Additional file 2: Ethics Committee. (PDF 176 kb)

## Abbreviations

95% CI: 95% confidence intervals
ADL: Activities of daily living
BP: Bodily Pain
FIM scale: Functional Independence Measure scale
GH: General Health
HRQoL: Health-related quality of life
IIDs: Individuals with intellectual disability
MH: Mental Health
PF: Physical Functioning
PSS: Perceived social support
RE: Role Emotional
RP: Role Physical
SD: Standard deviation
SF: Social Functioning
SF-36: Short Form**-**36 Health Survey
VT: Vitality
