# Supplementary material for: Do health and social support and personal autonomy have an influence on the health-related quality of life of individuals with intellectual disability?
Source: BMC Health Serv Res. 2019 Jan 23;19:63. doi: 10.1186/s12913-018-3856-5 (PMC6345008; doi:10.1186/s12913-018-3856-5)
Supplement: Supplementary file 1 — Statement of parental/legal guardian consent to participate (PDF 170 kb) [file 12913_2018_3856_MOESM1_ESM.pdf]

ASPRODES
Plena inclusión
Castilla y León

D. JUAN ANTONIO RECIO MOMPÓ, con D.N.I.: 12.367.991-V, domiciliado en Salamanca, c/ Mariseca, nº 1, como Gerente de ASPRODES con C.I.F.: G-37021946,

CERTIFICA

Que en la sede de la institución se ha llevado a cabo una reunión entre el Equipo de Investigación de la Universidad de Salamanca que dirige el profesor Mirón y los padres y tutores legales de las Personas con Discapacidad Intelectual institucionalizadas en ASPRODES-FEAPS con la finalidad de explicar el objetivo del Proyecto de investigación sobre Calidad de Vida en Personas con Discapacidad Intelectual y, solicitar el ***consentimiento informado*** para la participación de sus hijos y personas tutorizadas en el mismo y, que se les pueda realizar las entrevistas personales relacionadas con dicho proyecto a los largo de los próximos meses del 2015 y principios del 2016.

Lo que se hace constar a los efectos oportunos de investigación y aspectos éticos relacionados con la elaboración y procesamiento de la información.

Salamanca, a 22 de septiembre de 2015

Salarca
COOPERATIVA
C/ Mariseca nº1
37003 Salamanca
Teléfono 923 18 68 93
salarca@asprodes.es
www.arcagrupo.es

Utilidad Pública 26 marzo 1993 - C.I.F.: G-37021946
