# Supplementary material for: Do health and social support and personal autonomy have an influence on the health-related quality of life of individuals with intellectual disability?
Source: BMC Health Serv Res. 2019 Jan 23;19:63. doi: 10.1186/s12913-018-3856-5 (PMC6345008; doi:10.1186/s12913-018-3856-5)
Supplement: Supplementary file 2 — Ethics Committee. (PDF 176 kb) [file 12913_2018_3856_MOESM2_ESM.pdf]

HOSPITAL
UNIVERSITARIO
D SALAMANCA
Paseo de San Vicente, 58-182
37007 Salamanca
Comité Ético de Investigación Clínica
Teléfono: 923 29 11 00 – Ext 55 515

Sacyl
SANIDAD DE CASTILLA Y LEÓN

E-mail: comite.etico.husa@saludcastillayleon.es

TO WHOM IT MAY CONCERN

Dr. Ignacio DAVILA GONZÁLEZ, Secretary of the Clinical Research Ethics Committee for Clinical Investigation of the University Hospital of Salamanca

CERTIFIES THAT:

The research investigation project entitled: "¿INFLUYE EL APOYO SOCIAL PERCIBIDO Y LA AUTONOMÍA PERSONAL EN LA CALIDAD DE VIDA RELACIONADA CON LA SALUD DE LAS PERSONAS CON DISCAPACIDAD INTELECTUAL?" presented by Dr. JOSÉ ANTONIO MIRÓN CANELO, was submitted to our Ethics Committee.

Salamanca, April 11th 2016

COMPLEJO UNIVERSITARIO DE SALAMANCA
COMITÉ DE ÉTICA DE LA INVESTIGACIÓN CON MEDICAMENTOS

Dr. Ignacio DÁVILA GONZÁLEZ
Secretary of the Ethics Committee
